# Supplementary material for: Chimeric Measles Virus (MV/RSV), Having Ectodomains of Respiratory Syncytial Virus (RSV) F and G Proteins Instead of Measles Envelope Proteins, Induced Protective Antibodies against RSV
Source: Vaccines (Basel). 2021 Feb 16;9(2):156. doi: 10.3390/vaccines9020156 (PMC7920054; doi:10.3390/vaccines9020156)
Supplement: Supplementary file 1 [file vaccines-09-00156-s001.pdf]

Supplemental figure 1. Construction strategy for RSV F ectodomain fused to TM-CT domain of measles AIK-C. strain.

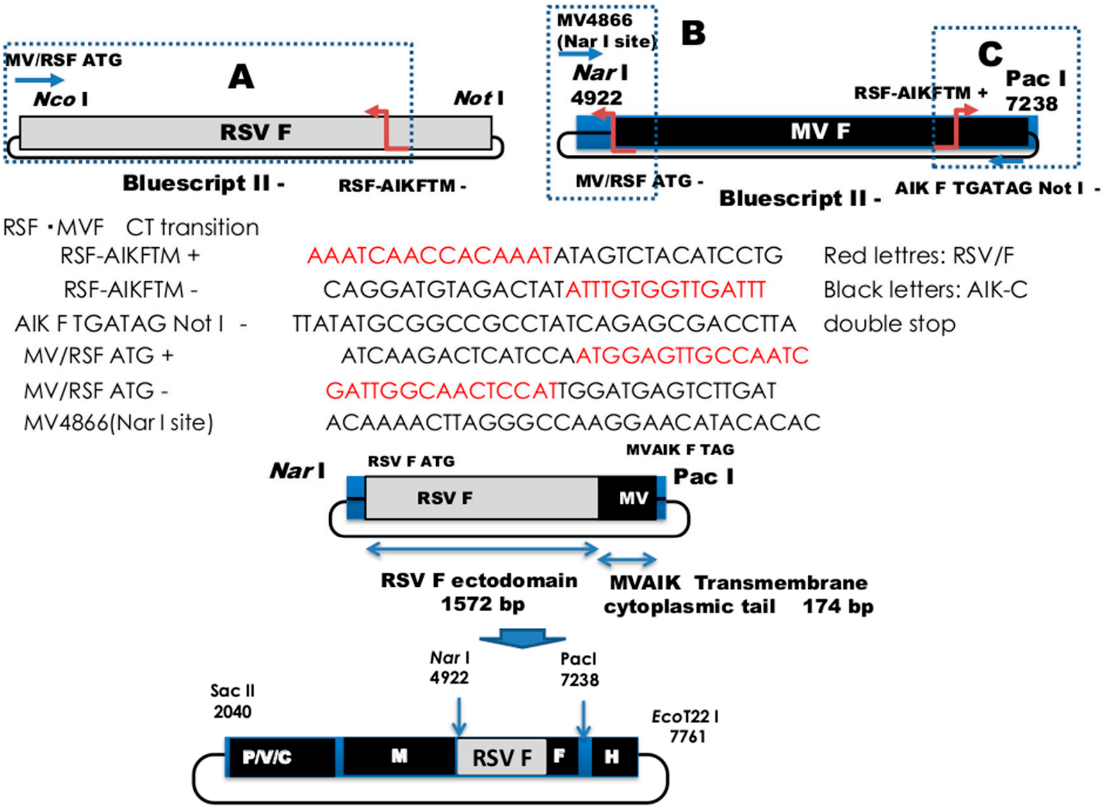

PCR was conducted in three parts.

A: From ATG of RSV F to RSV F ectodomain amplified by PCR with primer set of MV/RSF ATG+ and RSF-AIK F TM-, B: From MV4866 genome position of AIK-C to RSV F ATG amplified by PCR with MV4866 and MV/RSF ATG- primers, C: From TM-CT of measles AIK-C to MV stop region using RSF-AIK TM+ and AIK F TGA TAG *Not* I- primers. Three fragments were fused by PCR using MV4866 and AIK F TGA TAG *Not* I-primers. PCR product was cut by *Nar* I and *Not* I and inserted into MVAIK cloning plasmid from 2040 genome position (*Sac* I) to 7761 genome position (*Eco*T22I).

Supplemental figure 2. Construction strategy for RSV G ectodomain fused to TM-CT domain of measles AIK-C.

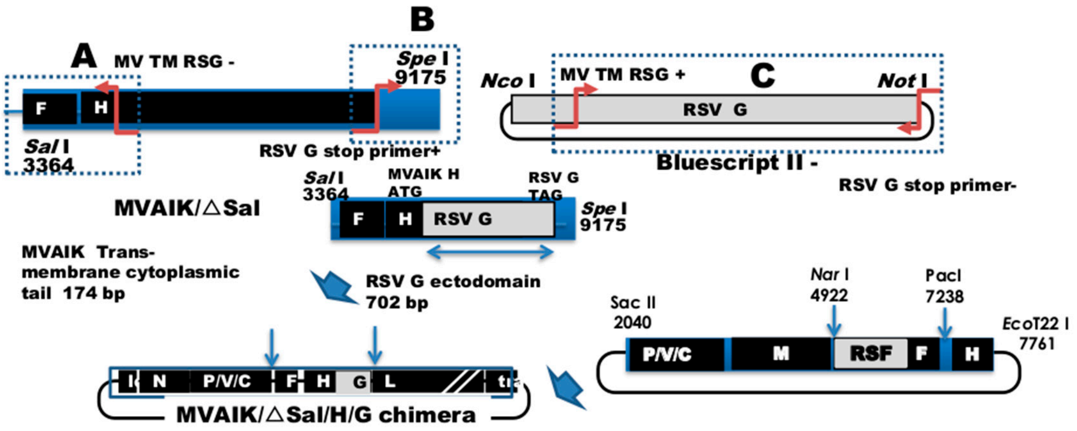

MVH CTTM, RSG ectodomain transition

MV TM RS G-	TGTGACTTTGTGAATGCCTGCAAT	Red letters: RSV/F
MV TM RSG +	ATTGCAGGCATTCACAAAGTCACA	Black letters: AIK-C
RSV G stop primer+	AACGAGTAGTGAGGCTGCTAGTGA	double stop
RSV G stop primer-	TCACTAGCAGCCTCACTACTCGTT	

PCR was performed in three parts.

A: From 3364 genome position (*Sal* I) to MV TM domain amplified by a primer set of MV3364 and MV TM RS G- primers, B: From RSV G stop region to measles 9175 genome position (*Spe* I) by a primer set of primers RSV G stop primer+ and measles 9175 primer, C: RSV G ectodomain using MV TM RSG+ and RSV G stop primer- . Three PCR fragments were fused by PCR using 3364 genome position (*Sal* I) and 9175 genome position (*Spe* I) primers. Chimeric DNA was inserted between *Sal* I and *Spe* I sites of MVAIK deletion Sal plasmid and this plasmid was used for full-length plasmid construction by inserting the plasmid fragment cut by *Sac* I and *Pac* I.

Western blotting analysis

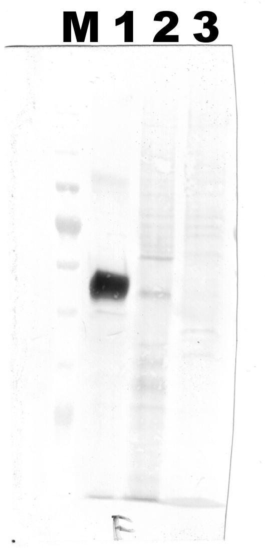

M: protein MW marker

1 : RSV

2 : MV/RSV chimera

3 : MVAIK

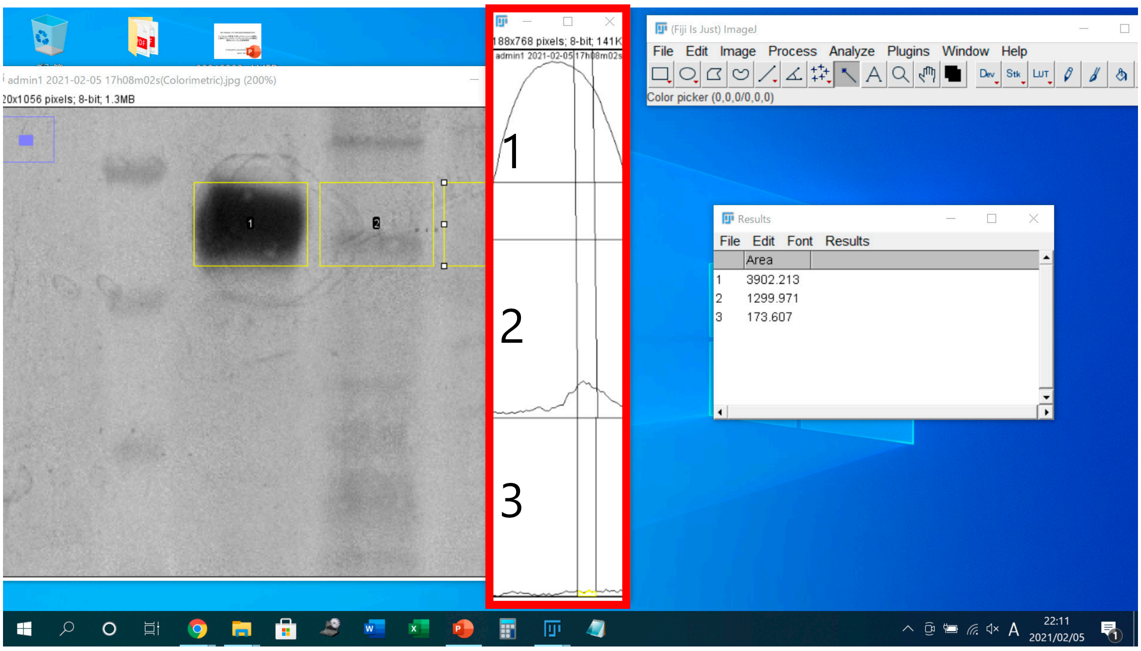

Squares of yellow line were analyzed for lanes 1, 2, and 3. The results of histogram of areas of 1, 2, and 3.

Right panel shows the results of Image J analysis.

	Area
1	3902.213
2	1299.971
3	173.607
